# Japan society of clinical oncology/Japanese society of medical oncology-led clinical recommendations on the diagnosis and use of tropomyosin receptor kinase inhibitors in adult and pediatric patients with neurotrophic receptor tyrosine kinase fusion-positive advanced solid tumors, cooperated by the Japanese society of pediatric hematology/oncology

**DOI:** 10.1007/s10147-019-01610-y

**Published:** 2020-01-24

**Authors:** Yoichi Naito, Saori Mishima, Kiwamu Akagi, Ataru Igarashi, Masafumi Ikeda, Susumu Okano, Shunsuke Kato, Tadao Takano, Katsuya Tsuchihara, Keita Terashima, Hiroshi Nishihara, Hiroyki Nishiyama, Eiso Hiyama, Akira Hirasawa, Hajime Hosoi, Osamu Maeda, Yasushi Yatabe, Wataru Okamoto, Shigeru Ono, Hiroaki Kajiyama, Fumio Nagashima, Yutaka Hatanaka, Mitsuru Miyachi, Yasuhiro Kodera, Takayuki Yoshino, Hiroya Taniguchi

**Affiliations:** 1 Department of Breast and Medical Oncology, National Cancer Center Hospital East, 6-5-1 Kashiwanoha, Kashiwa, Chiba 277-8577 Japan; 2 Saitama Cancer Center, Saitama, Japan; 3 Yokohama City University School of Medicine, Yokohama, Japan; 4 Juntendo University, Tokyo, Japan; 5 Tohoku University, Sendai, Japan; 6 National Center for Child Health and Development, Tokyo, Japan; 7 Keio University, Tokyo, Japan; 8 Tsukuba University, Ibaraki, Japan; 9 Hiroshima University Hospital, Hiroshima, Japan; 10 Okayama University, Okayama, Japan; 11 Kyoto Prefectural University of Medicine, Kyoto, Japan; 12 Nagoya University Hospital, Nagoy, Japan; 13 National Cancer Center Hospital, Tokyo, Japan; 14 Jichi Medical University, Tochigi, Japan; 15 Kyorin University Faculty of Medicine, Tokyo, Japan; 16 Hokkaido University Hospital, Sapporo, Japan; 17 Japan Society of Clinical Oncology (JSCO), Tokyo, Japan; 18 Japanese Society of Medical Oncology (JSMO), Tokyo, Japan; 19 Japanese Society of Pediatric Hematology/Oncology (JSPHO), Tokyo, Japan

**Keywords:** *Neurotrophic receptor tyrosine kinase* (*NTRK*) fusion, Tropomyosin receptor kinase (TRK) inhibitor, Advanced solid tumor, Tumor-agnostic treatment, Clinical practice guideline

## Abstract

**Background:**

The development of novel antitumor agents and accompanying biomarkers has improved survival across several tumor types. Previously, we published provisional clinical opinion for the diagnosis and use of immunotherapy in patients with deficient DNA mismatch repair tumors. Recently, efficacy of tropomyosin receptor kinase inhibitors against *neurotrophic receptor tyrosine kinase (NTRK)* fusion gene-positive advanced solid tumors have been established as the second tumor-agnostic treatment, making it necessary to develop the guideline prioritized for these patients.

**Methods:**

Clinical questions regarding medical care were formulated for patients with *NTRK*-positive advanced solid tumors. Relevant publications were searched by PubMed and Cochrane Database. Critical publications and conference reports were added manually. Systematic reviews were performed for each clinical question for the purpose of developing clinical recommendations. The committee members identified by Japan Society of Clinical Oncology (JSCO) and Japanese Society of Medical Oncology (JSMO) voted to determine the level of each recommendation considering the strength of evidence, expected risks and benefits to patients, and other related factors. Thereafter, a peer review by experts nominated from JSCO, JSMO, and Japanese Society of Pediatric Hematology/Oncology, and the public comments among all Societies’ members was done.

**Results:**

The current guideline describes 3 clinical questions and 15 recommendations for whom, when, and how *NTRK* fusion should be tested, and what is recommended for patients with *NTRK* fusion-positive advanced solid tumors.

**Conclusion:**

In the *NTRK* guideline, the committee proposed 15 recommendations for performing *NTRK* testing properly to select patients who are likely to benefit from tropomyosin receptor kinase inhibitors.

**Electronic supplementary material:**

The online version of this article (10.1007/s10147-019-01610-y) contains supplementary material, which is available to authorized users.

## Introduction

Historically, cancer care has been conducted based on the multifaceted evaluation of a case, such as the pathological diagnosis and staging of the disease, benefits and risks of treatments, and the patient's preference. The identification of the primary site and determination of histological type are important clinical information that forms the basis for determining treatment strategy. A recent advance in molecular biology has revealed the various biological characteristics of tumors and has enabled clinical development of tumor-agnostic drugs beyond the organ specificity of diseases.

In tumor-agnostic therapy, drugs are selected on the basis of biology beyond the primary site and type of cancer. In December 2018, in Japan, an anti-programmed cell death protein 1 (PD-1) antibody drug, pembrolizumab, was approved for advanced/recurrent deficient DNA mismatch repair (dMMR) solid cancers. This is the first drug in Japan for tumor-agnostic indications. Moreover, the efficacy of tropomyosin receptor kinase (TRK) inhibitors against *neurotrophic receptor tyrosine kinase (NTRK)* fusion gene-positive advanced solid cancers was demonstrated, and the U.S. Food and Drug Administration (FDA) approved larotrectinib in November 2018 and entrectinib in August 2019. Larotrectinib was also approved by European Medicines Agency (EMA) in September 2019. In Japan, entrectinib was approved in June 2019, which was earliest in the world. Entrectinib was the second tumor-agnostic drug approved in Japan.

The present guidelines systematically describe the items to be considered when selecting tumor-agnostic drugs including the timing and methods of testing, the positioning of each drug, and clinical care systems.

This article is a summary of the part describing *NTRK* in "Clinical Practice Guidelines for Tumor-Agnostic Treatments in Adult and Pediatric Patients with Advanced Solid Tumors toward Precision Medicine (*in Japanese*)". The part regarding dMMR has already been reported elsewhere [1].

The present guidelines provide a guide to diagnosis and treatment and should be utilized in clinical practice according to the recommendation levels described and by adjusting them for individual patients. They are expected to contribute to improving treatment outcomes in patients with solid cancer by utilizing them to perform appropriate tests and treatments on appropriate patients at appropriate timing.

## Materials and methods

The current guidelines systematically describe items to be considered when treating patients with *NTRK* fusion-positive solid tumors, including the timing and methods of testing *NTRK* fusions, as well as the positioning of TRK inhibitor therapy. In the clinical setting in Japan, if appropriate tests are performed on appropriate patients and the patients receive appropriate treatment at appropriate timing based on the recommended levels described in the present guidelines, treatment outcomes in patients with solid tumors are expected to be improved.

In the preparation of the guidelines, clinical questions (CQs) were formulated, and evidence for recommendation to each CQ was gathered by literature search for PubMed and Cochrane database (from January 1980 to August 2019). Moreover, critical publications and presentations in the international conferences not included above were added manually. Each search term and result for literature search appeared in each CQ. Based on the systematic review conducted according to the collected evidence, the committee members voted to determine a recommended level for each CQ (Table 1). The recommended levels were determined according to the strength of evidence for each CQ, potential benefit, demerit of patients, and other factors. In voting, whether the contents of medical care (including tests and indications) are approved or covered by health insurance in Japan was not considered. However, relevant information was described in the remarks column as needed. The committee's opinions were determined in the following manner: (1) if strong recommendation (SR) accounted for at least 70% of the vote, the committee's opinion was SR; (2) if (1) was not met, but SR + recommendation (R) accounted for at least 70% of the vote, the committee's opinion was R; (3) if (1) or (2) was not met, but SR + R + expert consensus opinion (ECO) accounted for at least 70% of the vote, the committee's opinion was ECO; (4) if not recommended (NR) accounted for at least 50% of the vote, the committee's opinion was NR, irrespective of the results of (1)–(3); and if none of (1)–(4) was met, there was "no recommended level."

**Table 1:** Degrees of recommendation and decision criteria

Degree of recommendation	Decision criteria
Strong recommendation [SR]	There is sufficient evidence and the benefits of testing outweigh the losses for patients
Recommendation [R]	There is certain evidence, considering the balance between benefits and losses for patients
Expert consensus opinion [ECO]	A certain consensus has been obtained although evidence and information that shows patient benefits cannot be said to be sufficient
Not recommended [NR]	There is no evidence

At present, some recommendations for CQs are not based on sufficient strength of evidence. It is also possible that the accumulation of new evidence in the future will lead to substantial changes in the descriptions in the text and recommended levels.

## Results

### Neurotrophic receptor tyrosine kinase (*NTRK*)

The *NTRK* 1 gene was discovered in a gene transfer assay using colorectal cancer tissue and reported as a cancer gene, *OncB*, by Pulciani, Barbacid, et al. in 1982 [2]. *NTRK* gene family members known to date are *NTRK1–3* (Table 2). *NTRK1–3* encode tyrosine receptor kinases, tropomyosin receptor kinase (TRK) A, TRKB, and TRKC, respectively. TRKA is expressed in the nervous system and gets phosphorylated when neurotrophin nerve growth factor (NGF) binds to it [3, 4]. Known ligands are brain-derived neurotrophic factor (BDNF) and neurotrophin (NT)-4 for TRKB and NT-3 for TRKC. Although NT-3 binds to other TRKs, it has the highest affinity with TRKC. TRKA regulates pain and body temperature, TRKB controls movement, memory, emotion, appetite, and body weight, and TRKC affects proprioception. The binding of a ligand to TRK induces the autophosphorylation of intracellular tyrosine residues, which activates downstream pathways including the phospholipase C (PLC)-γ, mitogen-activated protein kinase (MAPK), and phosphoinositide 3-kinase (PI3K)/AKT pathways, resulting in the differentiation, survival, and proliferation of cells [5, 6].

**Table 2:** *NTRK* gene family

Gene	*NTRK 1*	*NTRK 2*	*NTRK 3*
Synonyms	MTC; TRK; TRK1; TRKA; TRK-A; p140-TRKA	OBHD; TRKB; TRK-B; EIEE58; GP145-TRKB	TRKC; GP145-TRKC; gp145(TRKC)
Locus	1q23.1	9q21.33	15q25.3
NCBI Entrez Gene	https://www.ncbi.nlm.nih.gov/gene/4914	https://www.ncbi.nlm.nih.gov/gene/4915	https://www.ncbi.nlm.nih.gov/gene/4916

Both NTRK and TRK are used to describe either the name of gene or protein; in the current guideline, we describe *NTRK* for gene name and TRK for protein

Among various alterations of the *NTRK* genes, missense variants of the *NTRK* genes and *NTRK fusion*s are important in terms of the treatment of malignant tumors.

### Alteration and amplification

The alteration of the *NTRK* genes has been reported in tumors such as colorectal cancer, lung cancer, malignant melanoma, and acute leukemia. However, TRK activity of these altered genes is similar to or lower than that of the wild type (Table S1) [5, 7, 8]. Although association between the alteration of the *NTRK* genes and the development of malignant tumors has not been elucidated, it has been reported that if a tumor has the alteration of the *NTRK* genes (such as solvent front mutation, gatekeeper mutation, and glycine mutation of Asp-Phe-Gly (DFG) at the beginning of the activation loop), it becomes resistant to TRK inhibitors, larotrectinib, and entrectinib (Table 3). Moreover, an *NTRK1* splice variant, TRKA III, and an inframe deletion mutant (ΔTRKA) were reported in neuroblastoma and acute myeloid leukemia. These alterations are tumorigenic [8, 9]. As for the association between the *NTRK* genes and diseases other than malignant tumors, congenital insensitivity to pain with anhidrosis type IV, a hereditary disease, has a pathological variant of the *NTRK1* gene. The amplification of the *NTRK* genes has been reported in tumors such as breast cancer, cutaneous basal cell cancer, lung cancer, and neuroblastoma. Although it has been reported that TRKA and TRKC expression in neuroblastoma indicate a good prognosis [10], its tumorigenicity or significance as a target of treatment has not yet been elucidated.

**Table 3:**
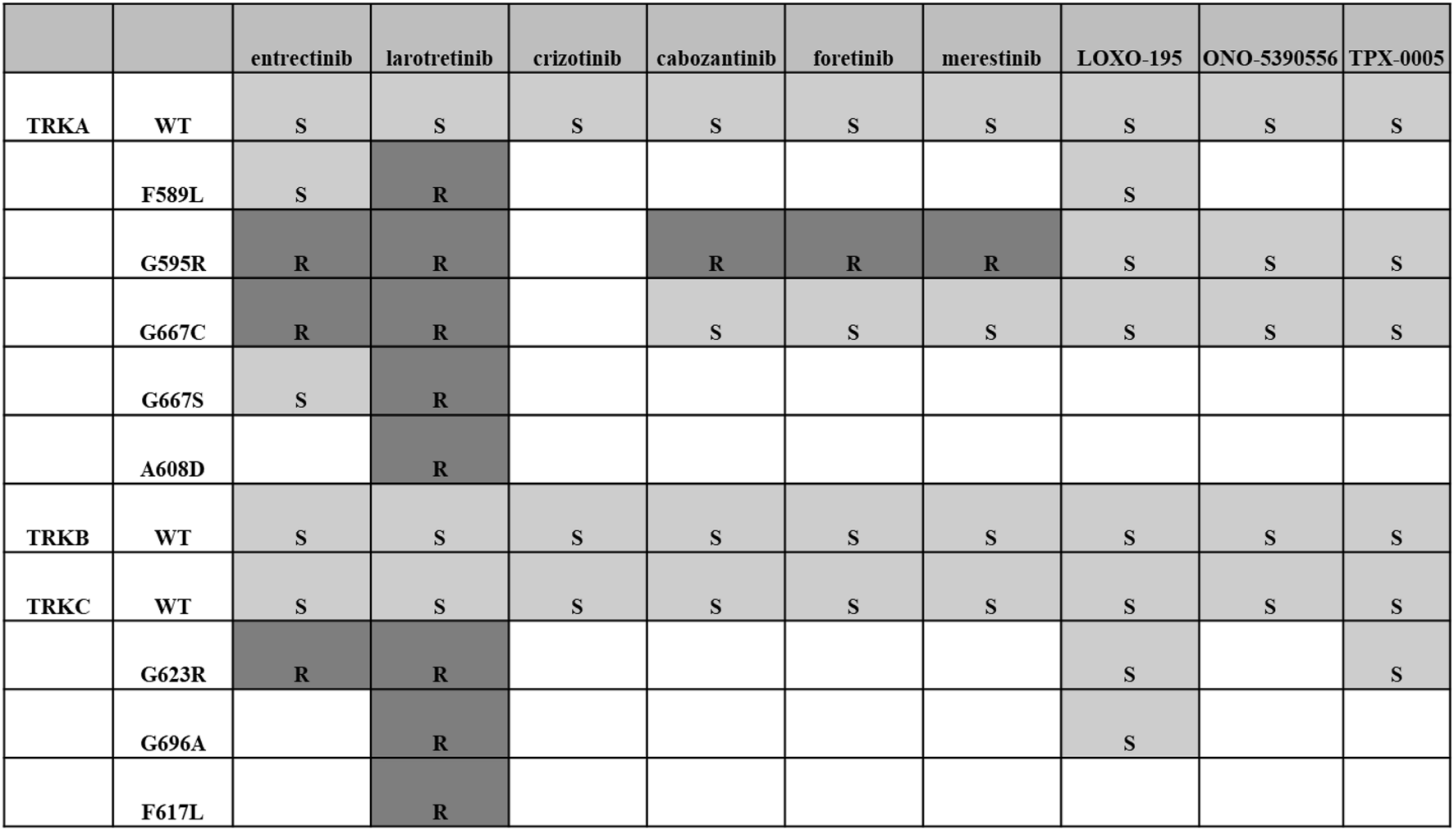
TRK inhibitors and resistant mutations

*WT* wild type, *S* sensitive, *R* resistant

### Rearrangement

*NTRK fusion*s (rearrangement) are tumorigenic genetic alterations reported in many cancer types [11]. Through intrachromosomal or interchromosomal translocation, a fusion gene is formed with a 3′ part of the *NTRK1–3* genes encoding the kinase region and a 5′ part of a partner gene (various genes have been reported). A ligand-independent kinase activation induced by the formation of a fusion gene is considered to contribute to carcinogenesis.

### Frequency of *NTRK* fusions by cancer type

*NTRK fusion*s are found in a wide variety of cancer types (Table 4) [12-15]. However, the frequency of *NTRK fusion*s is low in general, being 0.31% in the analysis result of The Cancer Genome Atlas (TCGA) database (*n* = 9966) [6]. On the other hand, there are rare cancer types in which *NTRK fusion*s are found at a high frequency, such as secretory carcinoma of the salivary gland (mammary analogue secretory carcinoma: MASC) [16, 17], secretory breast carcinoma [18-20], infantile fibrosarcoma (congenital fibrosarcoma) [21-24], congenital mesoblastic nephroma, and pediatric high-grade glioma (younger than 3 years old) [25].

**Table 4:** Reported frequency of NTRK fusion in various types of tumors

Tumor	Reported frequency	Frequency by TCGA database
Infantile fibrosarcoma (congenital fibrosarcoma)	90–100%	86–91%
Secretory breast carcinoma	80–100%	92%
Mammary analogue secretory carcinoma of the salivary gland	80–100%	93–100%
Congenital mesoblastic nephroma	83%	
Pediatric high-grade glioma	40% (< 3 years)	40% (< 3 years), 5.3%^a^
Melanoma	16% (Spitzoid tumors)	0.21% (1/476)
Cholangiocarcinoma	4%	
Gastrointestinal stromal tumor (GIST)	0.5–3%	
Inflammatory myofibroblastic tumor (IMT)	3%	
Thyroid cancer	2%	2.34% (12/513)
Colorectal cancer	1%	0.97% (3/310)
Sarcoma	1%	0.76% (2/263)
Head and neck squamous cell carcinoma	< 1%	0.38% (2/522)
Non-small cell carcinoma (NSCLC)	< 1%	0.18% (1/541)
Pancreatic adenocarcinoma	< 1%	0.56% (1/179)
Low-grade glioma		0.94% (5/534), 2.5% (3/120)^a^
Glioblastoma multiforme		0.56% (1/180)
Cervical cancer		0.33% (1/306)
Breast cancer		0.18% (2/1119)
Melanoma (pediatric)		11.11% (1/9)^a^
B-cell acute lymphoid leukemia		0.14% (1/716)^a^

^a^Data from St. Jude PeCan Data Portal (https://pecan.stjude.cloud/#!/about)

As for secretory carcinoma of the salivary gland (mammary analogue secretory carcinoma), Skalova et al. in the Czech Republic reported the presence of ETS translocation variant 6 (*ETV6*)*-NTRK3* fusion genes in tumors that developed in the salivary gland histologically resembling secretory breast carcinoma in 2010 [26]. It has been reported that MASC is more frequently found in men, and the mean age of onset is 44 years [27].

Secretory breast carcinoma is a very rare breast cancer; its frequency is < 0.15% among all breast cancers, with the median age of onset of 25 years, and it is found in both sexes [28]. Secretory breast carcinoma is triple-negative in many cases and has *ETV6-NTRK3* fusion genes. Although the prognosis is good, there have been reports of very late recurrence.

Infantile fibrosarcoma accounts for 12% of infantile malignant tumors. It has also been reported that 36–80% of infantile fibrosarcomas are congenital. It is rare that children 2 years of age or older develop infantile fibrosarcoma. Infantile fibrosarcoma frequently develops in limbs and has *ETV6-NTRK3* fusion genes. It has a better prognosis than adult fibrosarcoma. The efficacy of chemotherapy and cases of spontaneous regression has been reported [29].

Congenital mesoblastic nephroma is the most frequent renal tumor in infants 3 months of age or younger. It is recognized as a low-grade tumor with a good prognosis. It infrequently develops in both kidneys and is sometimes accompanied by hypercalcemia.

High-grade gliomas in children, particularly in infants younger than 3 years old, have better life prognoses than high-grade gliomas in older children and adults, and do not have alterations of the histone *H3.1* or *H3.3* gene, which are found in tumors in older children at a high frequency, or of the *isocitrate dehydrogenase* (*IDH*) *1* or *IDH2* gene, which are found in tumors in young adults at a high frequency. Recently, it has been reported that *NTRK fusion*s are found at a high frequency in tumors in infants [25].

As for lung cancer, in a study in 4872 patients at 7 institutions, *NTRK fusion*s were found in 11 patients (0.23%). Of them, six patients (55%) were male, eight patients (73%) were non-smokers/light smokers, and the median age was 47.6 years [30]. Nine of the 11 patients had adenocarcinoma. *NTRK fusion*s were also detected in squamous cell carcinoma and neuroendocrine carcinoma.

In most gastrointestinal stromal tumors (GISTs), genetic alterations of *KIT* or *platelet-derived growth factor A* (*PDGFRA*) gene that activate their functions are detected, while wild-type GIST, in which these genetic alterations are not detected, accounts for approximately 10% of all GISTs. *NTRK fusion*s are found in wild-type GISTs.

According to the Cancer Information Service ("Cancer Registration and Statistics in Japan" by the Cancer Information Service, National Cancer Center Japan; https://ganjoho.jp/public/index.html), the number of patients with breast cancer was 76,257 (women) in 2014, that with lung cancer 112,618, and that with colorectal cancer 134,453. Assuming that *NTRK fusion*s are found in 0.18% of breast cancers, 0.18% of lung cancers (non-small cell lung cancers), and 0.97% of colorectal cancers on the basis of data from the TCGA, the numbers of patients with *NTRK fusion*-positive breast cancer, lung cancer, and colorectal cancer are calculated to be 137, 202, and 1304, respectively, per year. Assuming that secretory breast carcinomas account for 0.15% of all breast cancers, the number of patients with secretory breast carcinomas is calculated to be 114 per year. It should be noted that, although the frequency of *NTRK fusion*-positive cancers is generally low among major cancers, the absolute number of *NTRK fusion*-positive major cancers is not small due to the high morbidity of major cancers even when compared to rare cancer types in which *NTRK fusion*s are found at a high frequency. At this point, there are no sufficient data to determine whether the frequency of *NTRK fusion*s differs between early cancers and advanced cancers. Further study on this issue is required in the future.

## NTRK testing methods

Methods for detecting *NTRK fusion*s include testing by next-generation sequencing (NGS) methods, reverse transcription polymerase chain reaction (RT-PCR), fluorescence in situ hybridization (FISH), and immunohistochemistry (IHC) [31, 32]. The NGS tests use not only DNA sequencing but also RNA sequencing. Most methods using DNA sequencing also analyze genetic alterations other than *NTRK fusion*s at the same time. In Japan, the OncoGuide™ NCC Oncopanel System and FoundationOne® CDx Cancer Genomic Profile have been approved for cancer genomic profiling. In addition to these, Oncomine™ Target Test and Todai OncoPanel are currently being used as advanced medical care. Some NSG tests are set to detect only known fusion partners and cannot detect unknown partners. They also have problems with repetitive regions and the tiling of entire introns. Therefore, it is suggested that they have lower sensitivity for detecting chromosomal translocation and inversion. There are some RNA sequencing methods that can detect *NTRK fusion*s irrespective of fusion partners. However, they have problems such that the use of panels specific to the fusion genes is required. FISH and RT-PCR have been used commonly for the detection in previous reports. However, these methods can analyze only single or a few genetic alterations. FISH can easily detect the presence of fusion genes irrespective of fusion gene partners, while RT-PCR cannot detect fusion gene partners other than known ones, which is a problem. Although IHC does not detect fusion genes themselves, there has been a report that when no TRK protein expression was detected by IHC using an antibody cocktail, no *NTRK fusion*s were found.[33] Therefore, the validity of IHC as a screening test is being examined. A gene expression analysis developed by NanoString Technologies, Inc. (hereinafter referred to as "NanoString") uses probes with unique molecular fluorescent barcodes that are specific to the sequences of target molecules. The probes are hybridized with target nucleic acid and then fixed on the surface of a cartridge. The sequence of the color barcodes bound to each target sequence is digitally counted using a fluorescent scanner. This gene expression analysis is expected to obtain good counting results of RNA samples prepared from formalin-fixed paraffin-embedded (FFPE) specimens. Since there are no sufficient data regarding the detection of *NTRK fusion*s, further studies are required in the future.

## TRK inhibitors

Examples of drugs with TRK inhibitory activity are shown in Table 5. Currently, the clinical development of entrectinib and larotrectinib is underway in Japan. Entrectinib is an oral tyrosine kinase inhibitor that inhibits ROS1, TRK, and ALK. At the European Society for Medical Oncology (ESMO) 2018 Congress, results from a pooled analysis of three studies in patients with *NTRK fusion*s, STARTRK-2, STARTRK-1, and ALKA-372–001 studies, were presented [34]. The response rate among 54 patients with soft tissue sarcoma, non-small cell lung cancer, secretory carcinoma of the salivary gland, and other tumors was 57.4%. Major adverse events included taste disorder (47.1%), constipation (27.9%), fatigue (27.9%), diarrhea (26.5%), peripheral edema (23.5%), dizziness (23.5%), and increased creatinine (17.6%). Entrectinib was approved for *NTRK fusion*-positive solid cancers by the FDA in August 2019, was granted PRIME (PRIority MEdicines) designation by the European Medicines Agency (EMA) in October 2017, and was also approved for *NTRK fusion*-positive advanced/recurrent solid cancers in Japan in June 2019.

**Table 5:** Examples of drugs with TRK inhibitory activity

	IC50 (nM)			Target other than TRK (IC50 < 500 nM)
	TRKA	TRKB	TRKC	
Entrectinib	2	0.1	0.1	ALK, ROS1
Larotrectinib	9	4	4	—
Cabozantinib	NA	7	NA	ALK, AXL, BLK, BTK, EPHA4, EPHB4, FAK, FLT1, FLT3, FLT4, FYN, KDR, KIT, LYN, MAP2K1, MET, PDGFRB, RAF1, RET, RON SAPK4, TIE2, YES
Crizotinib	1	1	NA	ABL, ALK, ARG, AXL, FES, LCK, LYN, MER, MET, RON, ROS1, SKY, TIE2, YES
Altiratinib	0.9	4.6	0.8	MET, TIE2 VEGFR2
Belizatinib	< 3	< 3	< 3	ALK
BMS-754807	7	4	NA	AURKA, AURKB, FLT3, IGF1R, INSR, MET, RON
Danusertib	31	NA	NA	ABL, AURKA, AURKB, AURKC, FGFR1, RET
DS-6051b	< 2	< 2	< 2	ALK, ROS1
LOXO-195	4	2	1	—
Merestinib	15–320	15–320	15–320	AXL, DDR1, DDR2, FLT3, MET, MERTK, MKNK1, MKNK2, MST1R, ROS1, TEK
MK-5108	2	13	NA	ABL, AURKA, AURKB, AURKC, AXL, BRK, EPHA1, EPHA2, FLT1, FLT4, GSK3A, JNK3, KDR, LOK, MER, PTK5, ROS, TIE2, YES
PLX-7486	< 10	< 10	< 10	AURKA, AURKB, CSF1R, MAP3K2, MAP3K3
Sitravatinib	5	9	NA	RET, CBL, CHR4q12, DDR, AXL, DDR1, DDR2, EPHA2, EPHA3, EPHA4, EPHB2, EPHB4, FLT1, FLT3, FLT4, KDR, KIT, MER, MET, PDGFRA, RET, RON, ROS, SRC

Larotrectinib is a selective oral TRK inhibitor. At the ESMO 2018 Congress, combined results from clinical studies in patients with *NTRK fusion*s, a phase 1 study in adults, a phase 1/2 study in children, and a phase 2 basket study, were reported [35]. Most of the patients had salivary gland tumor, soft tissue sarcoma, or thyroid cancer. The results of the pooled analysis of 109 patients showed the response rate of 81%. Major adverse events included fatigue, nausea, dizziness, vomiting, increased aspartate aminotransferase, and cough. Larotrectinib was approved by the FDA in November 2018. In July 2019, the Committee for Medicinal Products for Human Use (CHMP) issued a recommendation for conditional marketing authorization. In Japan, clinical trials are currently underway.

## Global status of approval of TRK inhibitors for patients with *NTRK* fusion-positive solid tumors (as of October 2019)

The approval status in Japan and by the FDA and EMA are shown in Supplemental Table S2.

## Recommendations in various guidelines

The descriptions of *NTRK fusion* testing and TRK inhibitors in the National Comprehensive Cancer Network (NCCN) guidelines are summarized in Table S3 (as of November 2019).

Among the ESMO guidelines, the 4th ESO-ESMO International Consensus Guidelines for Advanced Breast Cancer (ABC 4) state "If an ABC patient presents with a tumor with an *NTRK* fusion, treatment with a TRKi is a possible consideration." (Expert opinion/C).

The ESMO recommendations on the standard methods to detect *NTRK* fusions in daily practice and clinical research [36] propose the following algorithm (Fig. S1).

## Algorithm for NTRK testing

**Fig. 1:**
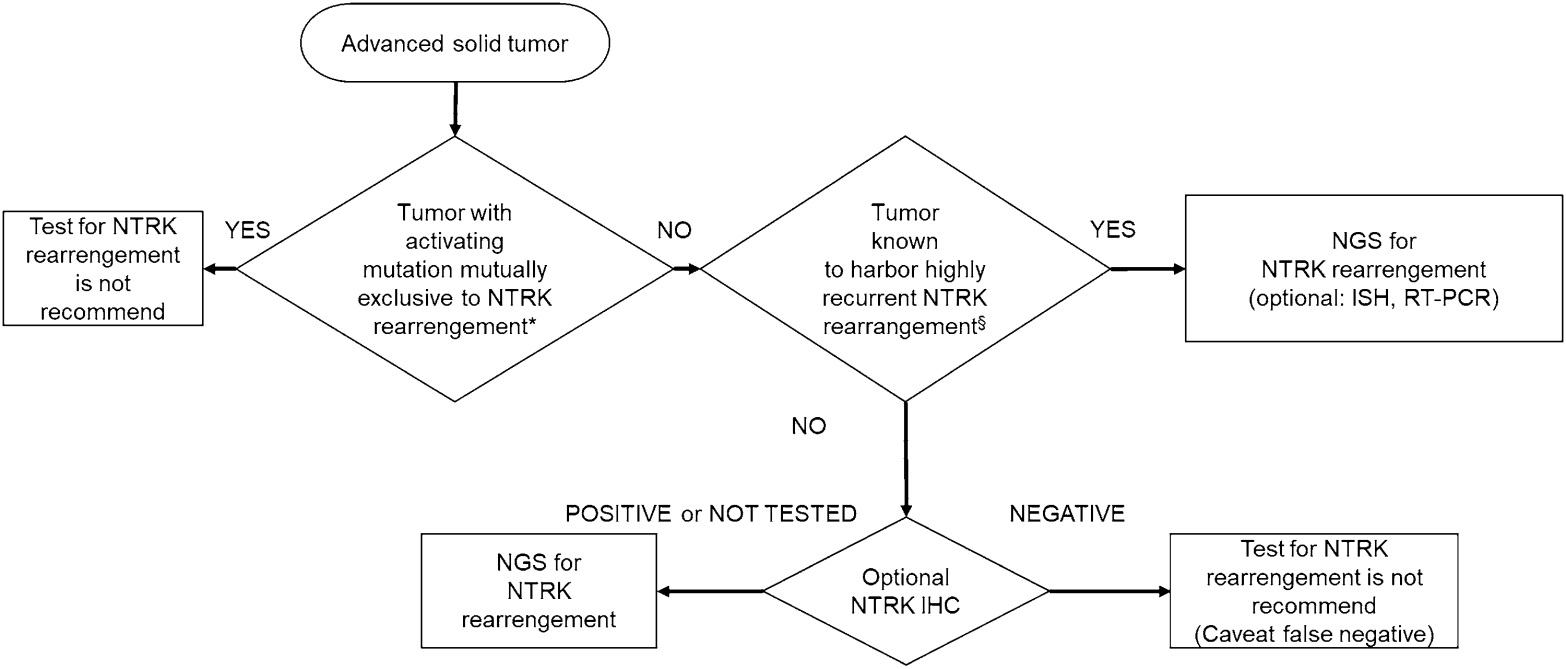
Algorithm for NTRK testing. §: Tumors such as secretory carcinoma of the salivary gland (mammary analogue secretory carcinoma), secretory breast carcinoma, infantile fibrosarcoma (congenital fibrosarcoma), congenital mesoblastic nephroma, and pediatric high-grade glioma (younger than 3 years old). *: Refer to CQ1. *NB* At this point, the optimal antibodies for TRK immunostaining have not been identified

Figure 1 summarizes the implementation of a rational approach for the detection of *NTRK1/2/3* fusions. To avoid the useless testing, we employed the algorithm of “mutually exclusive”; however, it should be noted that since the data regarding which mutation is mutually exclusive to *NTRK* fusions are limited, it is encouraged that the treating physician pay attention to the latest data.

## CQs (Table 6)

**Table 6:** Summary of recommendations

Recommendations	Level
CQ1: Targets of *NTRK* fusion testing	
CQ1-1. Is *NTRK* fusion testing recommended for patients with metastatic/recurrent solid cancers?	
1. *NTRK* fusion testing is not recommended for patients with solid cancers that have genetic alterations mutually exclusive with *NTRK* fusions	NR
2. *NTRK* fusion testing is strongly recommended for known cancer types in which *NTRK* fusions are detected at a high frequency	SR
3. *NTRK* fusion testing is recommended for all patients with metastatic/recurrent solid cancers other than those described above in order to determine the applicability of TRK inhibitors	R
CQ1-2. Is *NTRK* fusion testing recommended for patients with early solid cancers?	
1. *NTRK* fusion testing is recommended for patients with known cancer types in which *NTRK* fusions are detected at a high frequency even when their solid cancers can be radically treated	R
2. *NTRK* fusion testing is considered for all patients with early solid cancers other than those described above to determine the applicability of TRK inhibitors	ECO
CQ1-3. When should *NTRK* fusion testing be performed?	
It is strongly recommended that *NTRK* fusion testing should be performed before the start of the standard treatment or during the standard treatment	SR
CQ2: Testing methods for detecting *NTRK* fusions	
CQ2-1: Are NGS tests recommended for determining the applicability of TRK inhibitors?	
For determining the applicability of TRK inhibitors, NGS tests for which analytical validity has been established are strongly recommended	SR
CQ2-2: Are FISH and PCR recommended for the detection of *NTRK* fusions?	
1. FISH is not recommended as a screening test for *NTRK* fusions	NR
2. At this point, it is not possible to determine whether PCR is recommended as a screening test for *NTRK* fusions	None
3. Testing for *NTRK* fusions (particularly *ETV6-NTRK3* fusion genes) using FISH or PCR may be performed for known cancer types in which *NTRK* fusions are detected at a high frequency	ECO
CQ2-3: Is IHC recommended for the detection of *NTRK* fusions?	
1. IHC is recommended as a screening test for *NTRK* fusions	R
2. IHC is not recommended for determining the applicability of TRK inhibitors	NR
CQ3: Treatment for *NTRK* fusions	
CQ3-1: Are TRK inhibitors recommended for unresectable/metastatic/recurrent solid cancers possessing *NTRK* fusions?	
The use of TRK inhibitors is strongly recommended	SR
CQ3-2: When should TRK inhibitors be used?	
The use of TRK inhibitors from the initial treatment is recommended	R

*NR* Not recommended, *SR* Strong recommendation, *R* Recommendation, *ECO* Expert consensus opinion, *NGS* next-generation sequencing, *FISH*: fluorescence in situ hybridization, *PCR* polymerase chain reaction, IHC: immunohistochemistry

In recent years, clinical trials have reported the efficacy of TRK inhibitors for the treatment of advanced solid tumors with *NTRK* fusion gene-positive advanced solid tumors. In Japan, a TRK inhibitor in adult and pediatric patients with *NTRK* fusion-positive advanced solid tumors, regardless of the primary tumor site, has been approved. This has made it necessary to develop reference manuals, including guidelines, which enable smooth implementation of testing and treatment in the clinical setting. The clinical recommendations propose the following 15 requirements in 3 CQs regarding the *NTRK fusion* testing performed to select patients who are likely to benefit from TRK inhibitors.li

1. *NTRK fusion* testing is not recommended for patients with solid cancers that have genetic alterations mutually exclusive with *NTRK fusion*s.
2. *NTRK fusion* testing is strongly recommended for known cancer types in which *NTRK fusion*s are detected at a high frequency.
3. *NTRK fusion* testing is recommended for all patients with metastatic/recurrent solid cancers other than those described above to determine the applicability of TRK inhibitors.
4. *NTRK fusion* testing is recommended for patients with known cancer types in which *NTRK fusion*s are detected at a high frequency even when their solid cancers can be radically treated.
5. *NTRK fusion* testing is considered for all patients with early solid cancers other than those described above to determine the applicability of TRK inhibitors.
6. It is strongly recommended that *NTRK fusion* testing should be performed before the start of the standard treatment or during the standard treatment.
7. For determining the applicability of TRK inhibitors, NGS tests for which analytical validity has been established are strongly recommended.
8. FISH is not recommended as a screening test for *NTRK fusion*s.
9. At this point, it is not possible to determine whether PCR is recommended as a screening test for *NTRK fusion*s.
10. Testing for *NTRK fusion*s (particularly *ETV6-NTRK3* fusion genes) using FISH or PCR may be performed for known cancer types in which *NTRK fusion*s are detected at a high frequency.
11. IHC is recommended as a screening test for *NTRK fusion*s.
12. IHC is not recommended for determining the applicability of TRK inhibitors.
13. NanoString is not recommended as an *NTRK fusion* testing method for determining the applicability of TRK inhibitors.
14. The use of TRK inhibitors is strongly recommended.
15. The use of TRK inhibitors from the initial treatment is recommended.

Please keep in mind that these clinical recommendations will be revised in a timely manner, along with continuously and steadily advancing cancer treatment and new knowledge on biomarkers.

We will explain each CQ in detail.

## CQ1: targets of *NTRK fusion* testing

PubMed was searched with the keywords "NTRK or neurotrophic tropomyosin receptor kinase," "neoplasm," and "tested or diagnos* or detect*." The Cochrane Library was also searched with similar keywords. The time range of the search was from January 1980 to August 2019. From PubMed, 70 papers were extracted, and from the Cochrane Library, 1 paper was extracted. Four papers were added by manual search. By the primary screening, 68 papers were extracted, and by the secondary screening, 68 papers were extracted. These papers underwent a qualitative systematic review.
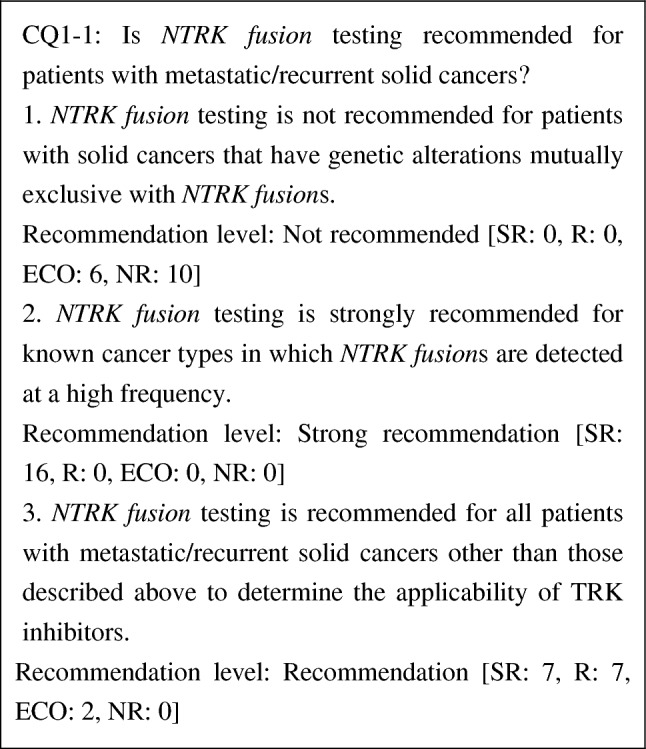


Clinical studies of entrectinib and larotrectinib, TRK inhibitors, have been conducted in patients with unresectable or metastatic solid cancers irrespective of the line of treatment and have demonstrated high efficacy. *NTRK fusion*s have been observed irrespective of cancer types, although at a low frequency. Moreover, no reliable biomarkers that can determine the presence or absence of *NTRK fusion*s in clinical settings have been established. Therefore, we strongly recommend the testing for all metastatic/recurrent solid cancers in which the presence of *NTRK fusion*s has been reported, to determine the applicability of TRK inhibitors [37]. We also strongly recommend the testing for tumors such as secretory carcinoma of the salivary gland (mammary analogue secretory carcinoma), secretory breast carcinoma, infantile fibrosarcoma (congenital fibrosarcoma), congenital mesoblastic nephroma, and pediatric high-grade glioma (younger than 3 years old), because *NTRK fusion*s (in particular *ETV6-NTRK3* fusion genes) are detected at a high frequency in these diseases. Because *NTRK fusion*s are mutually exclusive with other driver mutations, if mutually exclusive genetic alterations [e.g., *epidermal growth factor receptor* (*EGFR*) gene mutations, *anaplastic lymphoma kinase* (*ALK*) fusion genes, and *ROS1* fusion genes in non-small cell lung cancers; *rapidly accelerated fibrosarcoma* (*RAF*) gene mutations in malignant melanoma and colorectal cancer; and *KIT* gene mutations in GIST] of mitogenic pathways (groups of genes encoding the growth factor receptor, RAS, and MAPK pathways) are detected, a search for *NTRK fusion*s is not necessary.

During the voting, it was pointed out that whether testing is performed should be determined at the discretion of the attending physician and patient taking into account the cost and frequency.
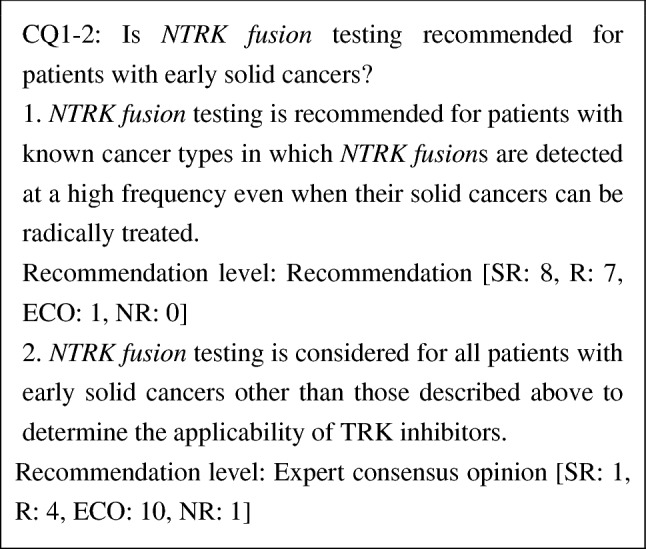


At present, the significance of TRK inhibitors as neoadjuvant/adjuvant therapy for patients with solid cancers possessing *NTRK fusion*s has not been established. However, in a phase 1 study of larotrectinib in pediatric patients [38], a partial response was obtained following the administration of larotrectinib in five patients and resection was subsequently performed. In three of them, tumors were completely resected. Because it has been reported that patients with metastatic/recurrent solid cancers possessing *NTRK fusion*s had a high response rate to TRK inhibitors, *NTRK fusion* testing is recommended for patients with known cancer types in which *NTRK fusion*s are detected at a high frequency. *NTRK fusion* testing may also be considered for radically treatable solid cancers other than the abovementioned types, taking into consideration the applicability of neoadjuvant therapy. If the use of TRK inhibitors is considered to reduce the long-term effects (late complications) of curative standard treatment particularly in children, the accumulation of long-term follow-up data of patients treated with TRK inhibitors is necessary, in addition to *NTRK fusion* testing.
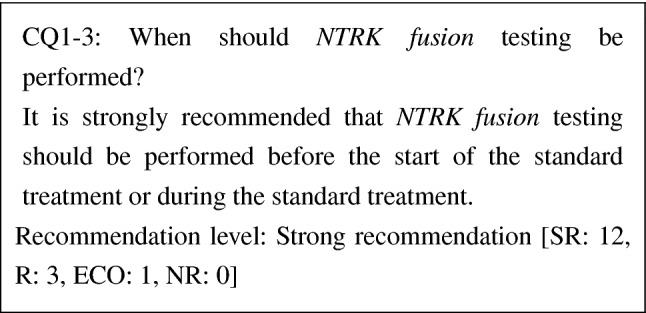


At this point, there has been no study report that compared the effectiveness of the standard treatment and that of TRK inhibitors in patients with metastatic/recurrent solid cancers possessing *NTRK fusion*s. The efficacy of TRK inhibitors was shown in the first line, and a high response rate has been reported. To prevent the loss of therapeutic opportunity for a patient who should be treated with TRK inhibitors because of the progress of the disease, we strongly recommend that *NTRK fusion* testing should be performed before the start of the standard treatment or during the standard treatment.

## CQ2: testing methods for detecting *NTRK fusion*s

PubMed was searched with the keywords "NTRK or neurotrophic tropomyosin receptor kinase," "neoplasm," "NGS," "In Situ Hybridization," "IHX," "NanoString," and "Polymerase Chain Reaction." The Cochrane Library was also searched with similar keywords. The time range of the search was from January 1980 to August 2019. From PubMed, 129 papers were extracted, and from the Cochrane Library, 5 papers were extracted. One paper was added by manual search. By the primary screening, 13 papers were extracted, and by the secondary screening, 13 papers were extracted. These papers underwent a qualitative systematic review.
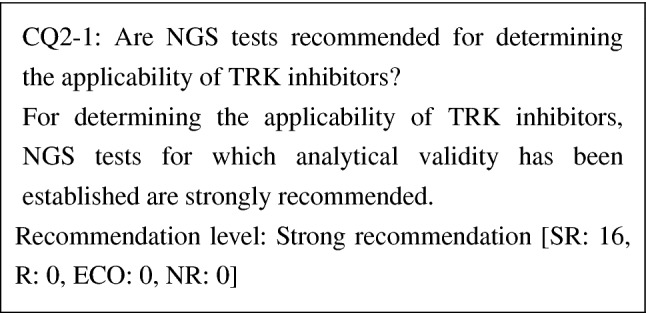


During the development of entrectinib and larotrectinib, various methods including NGS, FISH, and RT-PCR were used to determine the applicability of TRK inhibitors. Because reported *NTRK fusion*s vary over *NTRK1–3* genes and have various fusion partners, NGS tests that can detect fusion genes of all *NTRK1–3* genes are recommended. When a genetic test panel is used, it is necessary to check the range of *NTRK fusion*s the panel can detect. Some panels can only detect *NTRK fusion*s with known fusion partners, but other panels can detect *NTRK fusion*s irrespective of fusion partners. The analytical validity of tests is also important. In daily clinical practice, FFPE specimens are expected to be used. When fixing and storing specimens, and extracting DNA and RNA from them, it is desirable to follow guidelines established separately (Guidelines on the Handling of Pathological Tissue Samples for Genomic Research and Medicine, edited by the Japanese Society of Pathology).

As for the detection of *NTRK fusion*s, FoundationOne® CDx Cancer Genome Profile is approved as a companion diagnostic for entrectinib and can detect *NTRK1* fusion genes, *NTRK2* fusion genes, and *ETV6-NTRK3* fusion genes. Companion diagnostics for larotrectinib, which is approved in overseas countries, are being developed.

In both cases of a companion diagnosis and a comprehensive genetic analysis such as cancer genomic profiling, the use a test whose analytical validity has been established is recommended. In addition, because cancer genomic profiling, also examined factors other than *NTRK fusion*s, "Guidelines on the Development of Designated Core Hospitals for Cancer Genomic Medicine" (partially revised on July 19, 2019) and guidelines issued by relevant academic societies need to be referred to in the latter case.
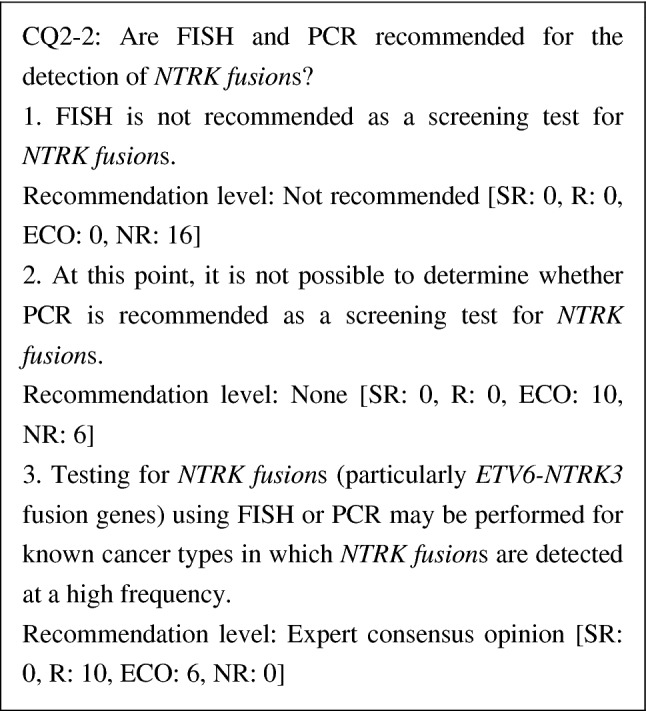


Because *NTRK fusion*s vary over *NTRK1-3*, FISH and PCR have limitations in detecting them. Although break-apart probes for *NTRK1-3* have been reported for FISH, performing three FISH assays in screening causes problems in cost and convenience. Regarding PCR, because the preservation of RNA in FFPE is problematic and the ranges of partner genes are unknown, it is not possible to judge what degree of detection accuracy can be ensured for PCR. Therefore, PCR cannot be recommended. However, if single gene tests that can solve these problems are developed, the PCR method needs to be reexamined. Although amplicon sequencing is based on the same principle as the PCR method, it can detect other genetic alterations and the detection accuracy has been specified. Therefore, amplicon sequencing will be discussed along with NGS.

Because almost all fusion genes detected in secretory carcinoma of the salivary gland (mammary analogue secretory carcinoma), secretory breast carcinoma, infantile fibrosarcoma (congenital fibrosarcoma), congenital mesoblastic nephroma, pediatric high-grade glioma (younger than 3 years old), etc., are *ETV6-NTRK3* fusion genes, the use of FISH or PCR may be considered.

In addition, it has been reported for other fusion genes that some of them cannot be detected by any of IHC, FISH, and NGS [39]. Therefore, careful attention should be paid to the false-positive and false-negative results of each testing method and close cooperation between clinicians and pathologists is required [40]. In particular, if *NTRK fusion*s are not detected in known cancer types in which *NTRK fusion*s are detected at a high frequency, it is desirable to confirm the results by other testing methods.

Conditions for approval of entrectinib include the provisions, "entrectinib should be administered to patients who are confirmed to be positive for the *NTRK fusion* by an experienced pathologist or testing facility. Approved in vitro diagnostics should be used for testing." Therefore, attention to these provisions is required. 
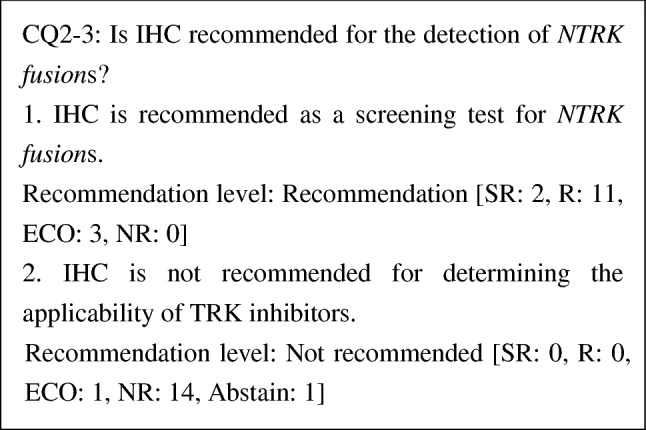


Although IHC is a method for detecting TRK protein, a positive IHC result does not mean the presence of an *NTRK fusion*. Therefore, IHC is not recommended as a test for determining the applicability of TRK inhibitors. However, there has been a report of a study using an antibody cocktail, in which *NTRK fusion*s were not detected when IHC was negative. Therefore, NGS or other tests can be omitted when IHC was negative, and IHC is expected to be valid as a screening test. It has been reported that in 33,997 patients, the sensitivity and specificity of DNA-based panel sequencing were 81.1% and 99.9%, respectively, and those of IHC (clone EPR17341) were 87.9% and 81.1% when an RNA-based panel (MSK-Fusion) was used as a control [41]. In this report, the sensitivity and specificity for sarcoma were not good, and the RNA-based panel was recommended. At this point, the optimal antibodies for IHC have not yet been identified, and sensitivity and specificity vary depending on the antibodies used. Therefore, care should be taken for false-positive and false-negative results when interpreting the test results. However, because test results can be obtained rapidly, further development in the future is expected.
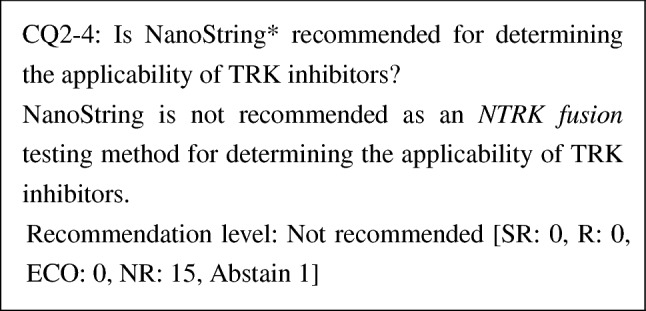


*: A gene expression analysis developed by NanoString Technologies, Inc. (referred to as "NanoString") uses probes with unique molecular fluorescent barcodes that are specific to the sequences of target molecules. The probes are hybridized with target nucleic acid and then fixed on the surface of a cartridge. The sequence of the color barcodes bound to each target sequence is digitally counted using a fluorescent scanner.

Because the validity of NanoString in detecting *NTRK fusion*s has not been demonstrated, NanoString is not recommended as a *NTRK fusion* testing method for determining the applicability of TRK inhibitors.

## CQ3: treatment for *NTRK fusion*s

PubMed was searched with the keywords "NTRK or neurotrophic tropomyosin receptor kinase," "neoplasm," "treatment," and "TRK inhibitor." The Cochrane Library was also searched with similar keywords. The time range of the search was from January 1980 to August 2019. From PubMed, 132 papers were extracted, and from the Cochrane Library, 6 papers were extracted. Two papers were added by manual search. By the primary screening, 38 papers were extracted, and by the secondary screening, 11 papers were extracted. These papers underwent a qualitative systematic review. 
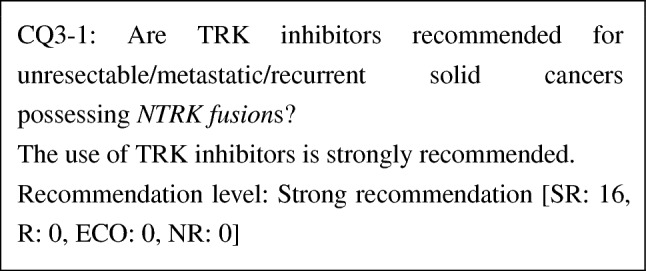


The efficacy of entrectinib and larotrectinib, TRK inhibitors, for solid cancers possessing *NTRK fusion*s has been demonstrated. Although studies comparing TRK inhibitors and other drugs have not been conducted at this point, response rates of TRK inhibitors are high. Moreover, adverse events by TRK inhibitors are mild in severity. Thus, the benefit of TRK inhibitors is considered to far outweigh the risk. It is also unlikely that the preference of patients varies. From these considerations, the use of TRK inhibitors is strongly recommended for solid cancers possessing *NTRK fusion*s.

At this point, no studies comparing the standard treatment and TRK inhibitors have been conducted. Therefore, if the standard treatment is available, whether a patient should be treated with the standard treatment or TRK inhibitors should be determined individually, taking into consideration anticipated effects, expected adverse events, and late toxicity of respective treatments. 
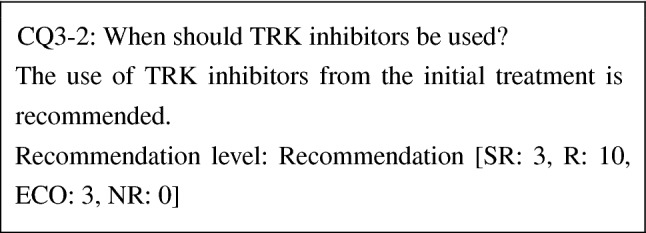


The efficacy of entrectinib, a TRK inhibitor, for solid cancers possessing *NTRK fusion*s has been demonstrated in patients who received the initial treatment, and, although studies comparing TRK inhibitors and other drugs have not been conducted, response rates of TRK inhibitors are high. Moreover, adverse events by TRK inhibitors are mild in severity. Thus, the benefit of TRK inhibitors is considered to far outweigh the risk. Therefore, the use of TRK inhibitors from the initial treatment is recommended.

At this point, no studies comparing the standard treatment and TRK inhibitors have been conducted. Therefore, if the standard treatment is available, whether a patient should be treated with the standard treatment or TRK inhibitors should be determined individually, taking into consideration the anticipated effects, expected adverse events, and late toxicity of respective treatments.

## Cost effectiveness

An agency that evaluates the cost effectiveness of certain drugs and reflects such information in determining whether the drug can be used in the public healthcare system (whether it should be covered by benefits) and in adjusting reimbursement prices (price control) is called a health technology assessment (HTA) agency. Many evaluations have been performed about the cost effectiveness of immune checkpoint inhibitors in patients with existing indications, i.e., non-small cell lung cancer, melanoma, kidney cancer, and other diseases by HTA agencies in many overseas countries. In Japan, where an HTA pilot program was introduced in 2016, the cost effectiveness data of nivolumab (Opdivo) and pembrolizumab (Keytruda) have been examined. As of September 2019, among TRK inhibitors, only entrectinib (Rozlytrek) is included in the National Health Insurance price list. However, entrectinib was not selected as the drug whose cost effectiveness data are to be submitted.

At this point, there has been no paper that has evaluated the cost effectiveness of entrectinib and larotrectinib, which are approved and unapproved drugs in Japan, respectively. As for evaluation by HTA agencies, the British NICE [42, 43] and the Canadian Agency for Drugs and Technologies in Health (CADTH) [44, 45] are currently evaluating these drugs, but at present, these evaluations have not yet been finalized. At any rate, the cost effectiveness evaluation is definitely important. In the future, the evaluation of the cost effectiveness of TRK inhibitors for *NTRK* fusion gene-positive patients is desired, as well as that of immune checkpoint inhibitors for MSI-H patients.

Genomic medicines that we have focused on in the present paper often target patients with diseases for which no other treatments exist. When evaluating such drugs, decision making based not only on the value of cost effectiveness (i.e., whether ICER is large or small) but also on the evaluation of other ethical/social factors and the effect on the entire finances is important. (Because an ICER value is independent of the number of patients, the effect on finances needs to be evaluated separately. This point is often misunderstood.) Previously, orphan drugs were outside the scope of cost effectiveness evaluation. However, extremely expensive therapeutic drugs [Kymriah, a chimeric antigen receptor T (CAR-T) cell therapy, and Zolgensma for spinal muscular atrophy] have become widely known and it has now become essential to quantitatively evaluate cost effectiveness for determining the values of such therapies. Unlike efficacy and safety data, it is essential to incorporate domestic data (particularly for costs) into cost effectiveness data. It is highly desired to perform a cost effectiveness evaluation using the data incorporating Japanese data at an appropriate time after the launch of a drug.

## Discussion

Regarding the cancer genome profile by NGS, the appropriate number of tests and the timing of the tests have not been determined by randomized controlled trials. However, as shown in the current guidelines, considering that tumor-agnostic treatment will become more widely used, it is strongly recommended that *NTRK* fusion be tested at the start of systemic therapy.

Regarding the number of tests, it is necessary to consider the purpose and implication of repeated tests and what method to use. It is known that specific *NTRK* mutations at the kinase domain bring resistance to TRK inhibitors. Therefore, *NTRK* mutations will be examined when the tumor which has been originally sensitive to TRK inhibitors become resistant following the administration of TRK inhibitors. It seems reasonable to consider second-generation TRK inhibitors which could overcome first-generation TRK inhibitor resistance; however, further investigation is needed.

Currently, investigation of mechanism for secondary resistance to certain molecular targeted therapy has been widely performed across cancers. From this point of view, it is not appropriate to limit the examination to only once in lifetime, but the appropriate timing and number of examinations are yet determined as they should be balanced with medical resources and costs.

Tumor-agnostic approach will become more common and the accompanying drug will be developed in near future. Potential examples of such tumor-agnostic targets other than *NTRK* include (but not limited to) *ALK*, *BRAF*, BRCAness, *FGFR*, *HER2*, *HER3,* homologous recombination deficiency (HRD), *KRAS*, *RET*, *ROS1*, tumor mutation burden (TMB) high.

## Conclusion

*NTRK* fusion is a rare but significant target for treatment across the tumor type. Clinicians must properly identify such rare but critical therapeutic targets to avoid missing the chance to provide therapeutic agents at the right time, through the right way, and to the right patients. In the *NTRK* guideline, the panel recommends the requirements for performing *NTRK* testing properly to select patients who are likely to benefit from TRK inhibitors.

## Electronic supplementary material

Below is the link to the electronic supplementary material.

Supplementary file1 (TIF 179 kb)

Supplementary file2 (DOCX 22 kb)
