# Supplementary figures and images for: Japan society of clinical oncology/Japanese society of medical oncology-led clinical recommendations on the diagnosis and use of tropomyosin receptor kinase inhibitors in adult and pediatric patients with neurotrophic receptor tyrosine kinase fusion-positive advanced solid tumors, cooperated by the Japanese society of pediatric hematology/oncology
Source: Int J Clin Oncol. 2020 Jan 24;25(3):403–17. doi: 10.1007/s10147-019-01610-y (PMC7046581; doi:10.1007/s10147-019-01610-y)

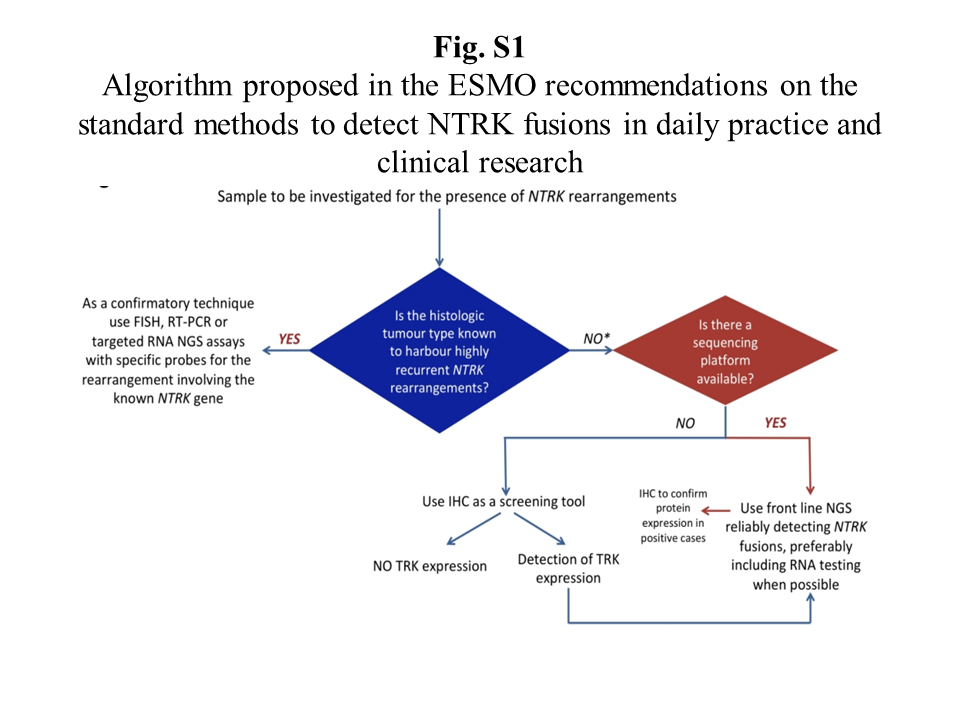

Supplement: Supplementary file 1 — Supplementary file1 (TIF 179 kb) [file 10147_2019_1610_MOESM1_ESM.tif]
