# Supplementary material for: Japan society of clinical oncology/Japanese society of medical oncology-led clinical recommendations on the diagnosis and use of tropomyosin receptor kinase inhibitors in adult and pediatric patients with neurotrophic receptor tyrosine kinase fusion-positive advanced solid tumors, cooperated by the Japanese society of pediatric hematology/oncology
Source: Int J Clin Oncol. 2020 Jan 24;25(3):403–17. doi: 10.1007/s10147-019-01610-y (PMC7046581; doi:10.1007/s10147-019-01610-y)
Supplement: Supplementary file 2 — Supplementary file2 (DOCX 22 kb) [file 10147_2019_1610_MOESM2_ESM.docx]

Supplemental materials

**Table S1. TRK activity of amino acid alteration by NTRK gene alteration**

| **NTRK** | **Tumor** | **Amino acid alteration** | **TRK kinase activity** |
| --- | --- | --- | --- |
| **NTRK1** | Malignant melanoma | M379I | Similar with wild-type |
|  |  | R577G | Similar with wild-type |
| **NTRK2** | Colorectal cancer | T695I | reduced activity |
|  |  | D751N | reduced activity |
|  | Lung cancer | L138F | Similar with wild-type |
|  | Malignant melanoma | P507L | Similar with wild-type |
|  | Lung cancer | M713I | reduced activity |
|  |  | R715G | reduced activity |
|  |  | R734C | reduced activity |

**Table S2. Approval status of TRK inhibitors in Japan and by FDA and EMA.**

| **Drug** | **Indication and dosage** |
| --- | --- |
| **Japan** |  |
| Entrectinib  (Rozlytrek®) | NTRK fusion gene-positive advanced / recurrent solid tumor  In general, for adults, 600 mg of entrectinib is orally administered once daily. The dose may be reduced according to the patient's condition.  Usually, children receive 300 mg / m2 (body surface area) orally once daily as entrectinib. However, it should not exceed 600 mg. The dose may be reduced according to the patient's condition. |
| **FDA** |  |
| Larotrectinib  (VITRAKVI®) | VITRAKVI is indicated for the treatment of adult and pediatric patients with solid tumors that:  •have a neurotrophic receptor tyrosine kinase (NTRK) gene fusion without a known acquired resistance mutation,  •are metastatic or where surgical resection is likely to result in severe morbidity, and  •have no satisfactory alternative treatments or that have progressed following treatment.  This indication is approved under accelerated approval based on overall response rate and duration of response. Continued approval for this indication may be contingent upon verification and description of clinical benefit in confirmatory trials.  **Recommended Dosage in Adult and Pediatric Patients with Body Surface Area of at Least 1.0 Meter-Squared**  The recommended dosage of VITRAKVI is 100 mg orally twice daily, with or without food, until disease progression or until unacceptable toxicity.  **Recommended Dosage in Pediatric Patients with Body Surface Area Less Than 1.0 Meter-Squared**  The recommended dosage of VITRAKVI is 100 mg/m2 orally twice daily, with or without food, until disease progression or until unacceptable toxicity. |
| entrectinib  (ROZLYTREK®) | **1.1 ROS1-Positive Non-Small Cell Lung Cancer**  ROZLYTREK is indicated for the treatment of adult patients with metastatic non-small cell lung cancer (NSCLC) whose tumors are ROS1-positive.  **1.2 NTRK Gene Fusion-Positive Solid Tumors**  ROZLYTREK is indicated for the treatment of adult and pediatric patients 12 years of age and older with solid tumors that:  •have a neurotrophic tyrosine receptor kinase (NTRK) gene fusion without a known acquired resistance mutation,  •are metastatic or where surgical resection is likely to result in severe morbidity, and  •have either progressed following treatment or have no satisfactory alternative therapy.  This indication is approved under accelerated approval based on tumor response rate and durability of response. Continued approval for this indication may be contingent upon verification and description of clinical benefit in the confirmatory trials.  **2.2 Recommended Dosage for ROS1-Positive Non-Small Cell Lung Cancer**  The recommended dosage of ROZLYTREK is 600 mg orally once daily with or without food until disease progression or unacceptable toxicity.  **2.3 Recommended Dosage for NTRK Gene Fusion-Positive Solid Tumors**  **Adults**  The recommended dosage of ROZLYTREK in adults is 600 mg orally once daily with or without food until disease progression or unacceptable toxicity.  **Pediatric Patients 12 Years and Older (Adolescents)**  The recommended dosage of ROZLYTREK is based on body surface area (BSA) as shown in TABLE 1 below. Take ROZLYTREK orally once daily with or without food until disease progression or unacceptable toxicity.  **Table 1: Dosing in Pediatric Patients 12 Years and Older (Adolescents)**   \| Body Surface Area (BSA) \| Recommended Dosage  (Orally once daily) \| \| --- \| --- \| \| Greater than 1.50 m2 \| 600 mg \| \| 1.11 to 1.50 m2 \| 500 mg \| \| 0.91 to 1.10 m2 \| 400 mg \| |
| **EMA** |  |
| larotrectinib  (VITRAKVI®) | VITRAKVI as monotherapy is indicated for the treatment of adult and paediatric patients with solid tumours that display a Neurotrophic Tyrosine Receptor Kinase (NTRK) gene fusion,  - who have a disease that is locally advanced, metastatic or where surgical resection is likely to result in severe morbidity, and  - who have no satisfactory treatment options  **Adults**  The recommended dose in adults is 100 mg larotrectinib twice daily, until disease progression or until unacceptable toxicity occurs.  **Paediatric population**  Dosing in paediatric patients is based on body surface area (BSA). The recommended dose in paediatric patients is 100 mg/m2 larotrectinib twice daily with a maximum of 100 mg per dose until disease progression or until unacceptable toxicity occurs. |

**Table S3. NCCN guideline recommendations for *NTRK* testing and TRK inhibitor for individual cancer type (As of Nov. 2019).**

| **Guideline** | **Version. Year** | **Recommendation for test** | **Recommendation for TRK inhibitor** |
| --- | --- | --- | --- |
| **Colon Cancer** | 3.2019 | Testing should include the neurotrophic receptor tyrosine kinase (*NTRK*) gene fusion | Larotrectinib or entrectinib is a treatment option for patients with metastatic colorectal cancer that is *NTRK* gene fusion positive |
| **Rectal Cancer** | 3.2019 | Testing should include the neurotrophic receptor tyrosine kinase (*NTRK*) gene fusion | Larotrectinib or entrectinib is a treatment option for patients with metastatic colorectal cancer that is *NTRK* gene fusion positive |
| **Small Bowel Adenocarcinoma** | 1.2020 |  | Larotrectinib is a treatment option for patients with metastatic SBA that is *NTRK* gene fusion positive |
| **Non-Small Cell Lung Cancer** | 7.2019 | Testing should include the neurotrophic receptor tyrosine kinase (*NTRK*) gene fusion | *NTRK* gene fusion discovered prior to first-line systemic therapy: Larotrectinib, Entrectinib  *NTRK* gene fusion discovered during first-line systemic therapy: Complete planned systemic therapy, including maintenance therapy, or interrupt, followed by larotrectinib or entrectinib |
| **Head and Neck Cancers** | 3.2019 | Check *NTRK* status for mammary analog secretory carcinoma (MASC) | *NTRK* therapy (eg, larotrectinib, entrectinib) for *NTRK* gene fusion-positive tumors |
| **Soft Tissue Sarcoma** | 4.2019 |  | Larotrectinib (for *NTRK* gene-fusion sarcomas)  Entrectinib (for *NTRK* gene-fusion sarcomas) |
| **Thyroid Cacinoma** | 2.2019 |  | Larotrectinib (for *NTRK* gene fusion-positive tumors)  Entrectinib (for *NTRK* gene fusion-positive tumors)  Larotrectinib and entrectinib are FDA approved for patients with *NTRK* gene fusion-positive advanced solid tumours. |
| **Occult Primary** | 1.2020 |  | Per physician discretion, TRK protein testing can be considered as part of broad immunohistochemistry testing (a positive test should then be confirmed with NGS):Drilon A, Laetsch TW, Kummar S, et al. Efficacy of larotrectinib in *TRK* fusion-positive cancers in adults and children. N Engl J Med 2018;378:731-739; Demetri GD, Paz-Ares L, Farago AF, et al. Efficacy and safety for entrectinib in patients with *NTRK* fusion-positive tumours: pooled analysis of STARTRK-2, STARTRK-1, and Alka-372-001. ESMO Meeting in Munich, Germany; October 12-23, 2018. Oral Presentation. |
| **Ovarian Cacner** | 2.2019 |  | Entrectinib or larotrectinib (for *NTRK* gene fusion-positive tumors) |
| **Pancreatic Adenocarcinoma** | 3.2019 | Useful in Certain Circumstances | Larotrectinib (If *NTRK* gene fusion positive) |
| **Cutaneous Melanoma** | 2.2019 |  | Larotrectinib for *NTRK* gene-fusion positive tumors |
